# Perspectives of Community- and Faith-Based Organizations about Partnering with Local Health Departments for Disasters

**DOI:** 10.3390/ijerph9072293

**Published:** 2012-06-28

**Authors:** Michael Stajura, Deborah Glik, David Eisenman, Michael Prelip, Andrea Martel, Jitka Sammartinova

**Affiliations:** 1 Department of Community Health Sciences, UCLA Fielding School of Public Health, Suite 26-081, Box 951772, Los Angeles, CA 90095, USA; Email: amartel@ucla.edu (A.M.); jitkasam@gmail.com (J.S.); 2 UCLA Health and Media Research Group, Department of Community Health Sciences, UCLA Fielding School of Public Health, Suite 26-081, Box 951772, Los Angeles, CA 90095, USA; Email: dglik@ucla.edu; 3 UCLA Center for Public Health and Disasters, UCLA Geffen School of Medicine, Suite 26-081, Box 951772, Los Angeles, CA 90095, USA; Email: deisenman@mednet.ucla.edu; 4 Department of Community Health Sciences, UCLA Fielding School of Public Health, Suite 26-078, Box 951772, Los Angeles, CA 90095, USA; Email: mprelip@ucla.edu

**Keywords:** disasters, public health emergencies, community-based organizations, faith-based organizations, local health departments

## Abstract

Public health emergency planners can better perform their mission if they develop and maintain effective relationships with community- and faith-based organizations in their jurisdictions. This qualitative study presents six themes that emerged from 20 key informant interviews representing a wide range of American community- and faith-based organizations across different types of jurisdictions, organizational types, and missions. This research seeks to provide local health department public health emergency planners with tools to assess and improve their inter-organizational community relationships. The themes identified address the importance of community engagement, leadership, intergroup dynamics and communication, and resources. Community- and faith-based organizations perceive that they are underutilized or untapped resources with respect to public health emergencies and disasters. One key reason for this is that many public health departments limit their engagement with community- and faith-based organizations to a one-way “push” model for information dissemination, rather than engaging them in other ways or improving their capacity. Beyond a reprioritization of staff time, few other resources would be required. From the perspective of community- and faith-based organizations, the quality of relationships seems to matter more than discrete resources provided by such ties.

## 1. Introduction

The contributions that community- and faith-based organizations make to disaster preparedness, response, and recovery are often praised and discussed. Their roles are important and well-established [1,2,3,4,5]. Local government agencies in the United States rightly acknowledge the value of such partnerships, but more can be done to improve their effectiveness. The value of good relationships with and among such community organizations is gaining attention. However, there have been few systematic inquiries into the dynamics of these relationships from the perspective of the community-and faith-based organizations themselves. The perspective that local health departments have of local community collaborations was documented by Varda *et al.* [6]. As formative research for part of a larger study, we sought to take a closer look at how community-based organizations (CBOs) and faith-based organizations (FBOs) perceive and relate to local health department (LHD) public health emergency planners (PHEPs). 

Two central tenets guided our inquiry. First, local government agencies can better serve the interests of their communities given healthy and effective relationships with CBOs and FBOs for disaster preparedness, response, and recovery. Second, the quality of these relationships is based on contextual, capacity, and personal characteristics of the local government agencies (with emphasis on local health departments). 

Moreover, it is important to acknowledge that LHDs have an excellent track record when it comes to partnering with CBOs and FBOs across a broad spectrum of public health programs [7,8,9]. However, this success in community partnerships for other programs and subjects does not necessarily seem to have been well translated to the emerging field of public health emergency planning. While this seeming discrepancy is not the basis of our study, it is important to frame our study in this manner. 

A disaster is an event (usually destructive) which strains or disrupts the social fabric and routines of a community and exceeds the inherent capacity of the affected community to respond to its effects [10,11,12]. Regardless of the jurisdiction’s size, a disaster forces a community-wide collaborative response that involves interagency coordination and the mobilization of non-governmental organizations that aid the community with disaster response, relief, and recovery services. We also include public health emergencies that can have a similar effect on a community, but they are not necessarily accompanied by physical destruction [13]. 

As have many other researchers, we faced the challenge of defining CBOs and FBOs. Although the definitions may seem self-evident, the reality is that many operational definitions have been used. A CDC workbook for public health and emergency management professionals provides a simple definition for CBOs: a “nonprofit that provides social services” [14]. Finding this definition lacking, we used the definition provided by the California Office of Emergency Services [15]. This definition acknowledges that CBOs are not necessarily nonprofit organizations, and it provides a description of the range of social services that they provide. This definition also highlights that CBOs might be local affiliates or chapters of national organizations, whereas others are more local or grassroots. We used this definition for the sake of consistency, but we acknowledge that it does not truly express the breadth and diversity of organizational types. We also included para-governmental organizations or programs which have characteristics in common with CBOs. These include local Community Emergency Response Teams (CERT) and the Medical Reserve Corps (MRC). 

The CDC workbook defined FBOs as “churches, synagogues, mosques, church sponsored service agencies, and all charitable organizations with religious affiliations” [14]. It is meaningful that this definition includes nonprofit organizations with a religious affiliation or inspiration. This coincides with an expanded definition found in Faith and Communities Engaged in Service (FACES) toolkit used by the Federal government’s Corporation for National and Community Service [16]. 

CBOs and FBOs are generally active in the disaster phases of preparedness, response, and recovery. Preparedness includes activities such as creating disaster plans, conducting training or educational activities, or collecting disaster supplies. Response activities are generally related to immediate disaster relief and mass care (e.g., water, food, and safe shelter), but in the case of some groups (notably CERT and MRC), they can include lifesaving activities (e.g., search and rescue, triage, and first aid or basic life support). 

Disaster recovery is acknowledged as the longest and most complex phase, and it is also where CBOs and FBOs can have the greatest differential impact. This is especially true with human recovery, which involves information and referral services, direct assistance to individuals and families in the form of donations or counseling, and financial support to help families reestablish their households [17]. Indeed, CBOs and FBOs fill a critical role with regard to human recovery for which government agencies are less well-suited or equipped, and thus government agencies often contract with CBOs/FBOs or delegate responsibility to them for these services because government agencies lack the staff or expertise to provide them. Human recovery services are also a good fit for CBOs/FBOs when there is a good mission fit and because they are often leading humanitarian organizations within the communities that they serve [14]. 

Moreover, these organizations may be better able to reach at-risk populations: the economically disadvantaged, groups with limited English proficiency or low literacy, those with certain medical issues or disabilities, or groups characterized by cultural, geographic, or social isolation [14]. Another critical factor is that CBOs and FBOs are recognized as trusted agents for the communities that they serve (in comparison to government agencies) [14]. Another reason that CBOs and FBOs are important is because they possess or have access to resources for the community (facilities, volunteers, a donor base, *etc*.). These resources can be critical to any local government agency’s efforts to help its community prepare for, respond to, and recover from a disaster. 

Because of this, many Federal programs encourage or even direct their local government counterparts to collaborate with CBOs and FBOs [14]. Government agencies at all levels have sought to organize and harness CBO/FBO disaster involvement due to historical examples of poorly coordinated disaster response and recovery efforts in the United States [5]. This collaborative philosophy is perhaps best expressed by the “Whole Community” approach used by the Federal Emergency Management Agency (FEMA). This philosophy stipulates as policy the values of collaboration, local empowerment, and collective community response to disaster [18]. At the highest level, the CDC and FEMA strongly encourage local government agencies to proactively collaborate with CBOs and FBOs. 

The mechanism by which this collaboration happens subsequently matters, as this process can influence the satisfaction and effectiveness associated with community disaster collaborations. The Voluntary Organizations Active in Disasters (VOAD) model has emerged as the most prolific means for encouraging the effective participation of CBOs and FBOs in disaster preparedness, response, and recovery [14]. In theory, VOADs (or equivalent organizations the serve the same functional/structural purpose) allow their member organizations to collaborate, coordinate, cooperate, and communicate more effectively. 

VOADs ideally provide their member and partner organizations with better cost-effectiveness when it comes to creating and maintaining relationships. This is because the member organizations can develop more and better relationships before a disaster rather than making new relationships under emergent conditions. The observation is that hastily-formed relationships in a moment of crisis lead to a variety of problems. 

There is emerging literature which examines the issue of emergent social networks among organizations responding to disasters [19,20,21,22,23]. However, fewer disaster studies have been able to examine the function and dynamics of pre-disaster social networks after disaster. Several studies discuss inter-organizational “linkages”, “ties”, “integration”, “coordination”, or even “partnerships” in the generic sense. Wineman *et al*. concluded that health centers’ lack of “community based planning leaves an already vulnerable population at greater risk” [24]. Lerner *et al*. describe effective partnership behaviors between local health departments and emergency care providers of seven model communities [25]. Paige *et al*. demonstrate how pandemic flu exercises improve local health department relationships with the community partners who participated [26]. Kapucu uses a similar argument, pointing out how communities repeatedly exposed to the threat of hurricanes benefit from improved relationships among local government agencies and CBOs/FBOs as a result of repeated and extensive planning activities (essentially, serving a function similar to exercises) [27]. 

Another Kapucu article explicitly uses Social Network Theory applied to the 9/11 terrorist attack in New York to demonstrate how nonprofit sector organizations (*i.e.*, CBOs) met gaps in disaster service delivery to the public that local government agencies were not able to fulfill [28]. He also presented an argument that these services provided by CBOs could have been improved via quantifiable social network improvements to the relationships between local government agencies and the CBOs. This article supports the logical foundation from which our inquiry begins. Additionally, Harris and Clements applied Social Network Theory when studying the degree to which public health emergency planners in Missouri were networked to each other (across counties), yet noted that local networking to CBOs and FBOs within their own counties might be lacking [29]. LHDs lacking sufficient ties within their own communities would have to rely on network intermediaries, as discussed by Lind *et al.* [30]. In this study, we will address the qualitative relationships with LHDs (as well as some other local government agencies) and how they are perceived by CBOs/FBOs as they relate to disasters and public health emergencies. 

## 2. Methods

### 2.1. Sample

We conducted 20 key informant interviews representing a wide array of CBOs/FBOs in the United States, most of which were explicitly active in disasters. The purposive sample included representatives from a variety of organizational types, sizes, missions, levels and types of activity, and jurisdictional characteristics. The respondents were from 14 different U.S. states. Seven respondents represented organizations that operated in four different major U.S. urban areas. One respondent also represented a CBO from a semi-autonomous Native American tribal jurisdiction in the U.S.

The goal was to establish an array of perspectives that included voices from dense urban areas to remote or rural areas, from local chapters of major national organizations to the truly “local” and grassroots/parochial organizations. Our sample included major nonprofit organizations (e.g., local American Red Cross chapters), small community-based organizations, faith-based organizations (e.g., local community places of worship), and faith-affiliated nonprofit groups (the counterparts of major faith-based groups, such as Catholic Charities or the Baptist Convention). Similarly, our sample included organizations that were part of locally established county-level VOADs (or equivalent organizations), as well as organizations where no such groups existed. 

Several of the organizations interviewed existed primarily for purposes other than disasters, but they include disaster preparedness, response/relief, or recovery within their scope. Also, in three of the 20 interviews completed, the explicit purpose of the organization was to serve as an intergroup networking hub or collaborative with respect to disaster activities in the area. Table 1 provides a description of the range of organizational and jurisdictional types represented in the sample of 20 organizations interviewed. 

**Table 1:** Characteristics of sampled organizations.

CBO/FBO Descriptor	n (out of 20)
Community-based organizations	11
Faith-based organizations	9
Local chapters of large-scale nonprofit organizations	10
Local organizations with no affiliation to larger organizations	10
Urban jurisdictions	10
Rural jurisdictions	10
Mission exclusive to disasters	9
Mission broader than but includes disasters	6
Mission not specific to disasters	5
Active member of a local VOAD (or equivalent)	12
No local VOAD (or equivalent) present	5
States represented in the sample	14

### 2.2. Data Collection

Each interview was conducted by telephone using a semi-structured interview guide approved by the UCLA Institutional Review Board. There were three sets of questions: organizational characteristics and direct disaster-related experiences or participation; external/intergroup relationships and dynamics; and, where relevant, an optional set of questions for organizations that served as or played a key leadership role within a local VOAD. 

The average interview time was 40 minutes. After transcription, each interview was coded by two team members (in mixed pairs) using *Atlas.ti*. Following convergence coding, all 20 interviews were analyzed for common themes. 

A qualitative analysis of the transcribed interviews was applied to identify and elucidate six key themes that emerged from the interviews. In some cases, common themes were identified across all groups. In others, certain themes emerged relative to organizational or jurisdictional characteristics. The viewpoints and concerns expressed by CBOs/FBOs as they relate to LHDs (and sometimes other local government agencies) led to novel findings not uncovered during the literature review (or expressed as conventional wisdom within the field). 

## 3. Results

We identified six critical themes from our analysis that expand the understanding of CBO/FBO relationships with LHDs in the context of public health emergency and disaster preparedness, response, and recovery. The following sections identify, illustrate, and explain each of these themes through the lens provided by our key informants. We placed emphasis on novel, counterintuitive, or interdisciplinary themes that expanded our understanding of LHD-CBO/FBO relationships as they relate to disasters and that move the conversation beyond what we learned from our literature review. In many cases, the respondents also referred to other CBOs/FBOs within their local network as well as their relationships to LHDs or other local government agencies. 

### 3.1. Partnership Facilitators and Barriers

The first key theme that emerged from our analysis across virtually all interviews dealt with facilitators and barriers to effective partnerships that centered on intergroup dynamics issues of perception, trust, transparency, expectation management, competing agendas, and “organizational egos” (regarding both local government agencies and other CBOs/FBOs). Respondents rarely identified separate and discrete lists of facilitators and barriers. Rather, the overwhelming majority pointed to the presence or absence of a particular relationship characteristic as a facilitator or a barrier (as one respondent described a relationship barrier, “the opposite of what I just told you” regarding something she had just identified as a relationship facilitator). 

To illustrate one example regarding trust and transparency, a CBO respondent spoke of how her local health department partner would regularly solicit her input at planning and coordination meetings. However, the LHD would later announce decisions or policies without explaining how or why the decision was reached, and the results seemed entirely separate from or contrary to the input provided by the CBO. The key issue for this respondent was the lack of transparency inside the “black box” where decisions were made, along with a lack of understanding regarding how her input was used in that decision making process. Her input was solicited as if her organization was a respected partner, but then the final outcome seemed otherwise. 

Another example pointed to issues of expectation management—the perception of whether or not a task or commitment was satisfied by all parties in a given interaction. A respondent who represented a local chapter of a faith-affiliated nonprofit organization described misunderstandings regarding the actual missions and capacities among the partner organizations. Organizational representatives (including the local health department) seemed to assume they knew what their partner organization was capable of providing. In nearly all cases where problems with expectation management came up, one organization would overestimate the capability of its partner for a specific task rather than underestimate it, and conflict or dissatisfaction emerged as a result of the perception that the partner organization was not contributing all that it could or should be doing. In contrast, CBOs/FBOs often reported that their capabilities were underestimated for issues where their help was not solicited. They felt they had more to offer in areas where they were not engaged, indicating untapped potential and benefits never realized by the LHD. 

Another nearly universal discussion point from the CBO/FBO representatives dealt with perceptions about power relations among CBOs/FBOs when it came to how they perceived their relationship with the LHD. Perceptions that they were not included or “at the table” for certain decisions undermined their satisfaction with their relationships with both the LHD and other local CBOs/FBOs. One CBO representative shared an excellent example of this phenomenon, reporting that the LHD met frequently with specific CBOs/FBOs but neglected to include or invite other partners. This CBO representative drew the conclusion that the LHD must have valued its relationship with her CBO less than the others, but she felt that her organization had at least as much to contribute as the others. 

### 3.2. Resources Matter, but Not as Much as We Might Think

The issue of constrained resources (e.g., funding, supplies and materiel, access to facilities, volunteers, training, or educational opportunities) was another theme that came up very often. This was presented in two contexts. First, the perceived value or prominence of a relationship was often based on the resources that an organization could either provide to or get from the LHD. Some CBOs/FBOs felt more valuable based on what they brought to the table, or they felt that they needed (or perhaps did not need) a good relationship with the LHD to secure discrete resources. In one example, a FBO reported that it felt marginalized because a local CBO dominated the relationship with the LHD since it had received its own Federal grant funding for disaster preparedness. 

Several CBOs/FBOs also stated that they did not want or need anything from their LHD other than a seat at the table, where they would be treated as partners. Quite surprisingly, several CBOs/FBOs suggested that they were not aware of any potential resources available through forming a relationship with their LHD. The notion of receiving resources as a function of their relationship was reported as irrelevant to their satisfaction with the relationship. Rather, the quality of their relationship with the LHD was less about discrete resources and more about access to staff, trust, and respect. One faith-affiliated nonprofit organization reported that they were self-sustaining in terms of resources, and they wanted nothing more than disaster area access to allow them to work (as their faith inspired them to do). Another faith-affiliated nonprofit organization said that they wanted to maintain their independence from relying on government organizations for resources. Their work was motivated by their faith, and if they received support from government agencies, it might negatively affect perceptions among their church members or their reputation with the community. Yet another CBO reported that it never occurred to them that they might be able to get resources from their local health department because of widespread reports of budget problems; the issue of receiving resources through their connection to the LHD was entirely irrelevant to them. Thus, these inter-organizational relationships seemed less centered on “what” (resources) and more focused on “how” (nature of interaction). 

### 3.3. Formal *vs.* Informal Relationships

Some CBOs/FBOs conveyed that they felt pressured to enter into formalized and/or written relationships with LHD partners who asked for Memoranda of Understanding (MOUs) as a preliminary or prerequisite step to proceeding with an inter-organizational relationship. MOUs are commonplace written agreements that outline how organizations relate to each other, and they can have liability, funding, or governance implications. In many cases, they are seen as desirable. 

While large, national CBOs/FBOs that are more professionalized and networked seemed more comfortable with MOUs as the basis for moving forward with a relationship, the smaller, more parochial, or grassroots CBOs/FBOs generally expressed resistance to signing a formal document between their organizations prior to establishing solid interpersonal relationships among organizational representatives. This was even more pronounced in rural areas. In short, they wanted to know who they are working with before signing what they essentially perceive as a contract, and they resisted the perceived pressure to sign [31]. LHDs’ perceived insistence on obtaining a MOU prior to establishing an interpersonal relationship often inhibited the development of good inter-organizational relationships. 

Other factors influence the willingness of CBOs/FBOs to sign MOUs. One FBO stated that they did not want to be in a “contractual” relationship with any local government organization, as it might be misperceived or mischaracterized by its members or clients. They wanted to remain independent of formal ties to any government agency. A CBO stated that they simply wanted to be “in contact with” the LHD, specifically so that they could stay informed and have an open line of communication, if needed. From their perspective, they did not want or need a formal, written agreement with the LHD to accomplish their goal in the relationship. 

### 3.4. Voluntary Organizations Active in Disasters (VOADs)

Several of the organizations sampled were leaders or active members of a local Voluntary Organizations Active in Disasters (VOAD) group; three of the respondents were actually paid employees of the local VOAD organization. We also found examples of local disaster organization networks or collaboratives that were *de facto* VOADs in the way that they operated, even though they did not self-identify as such. Again, a VOAD is essentially an organization or committee that provides a networking and coordination function for local CBOs, FBOs, and local government agencies that play a role in disasters at the local level [5]. 

One VOAD representative who we interviewed from a densely populated urban area leads a network with a tightly-knit core of CBO/FBO members who interact regularly. However, the respondent reported that overall membership seemed very low compared to the large number of CBOs/FBOs in the jurisdiction. Member recruitment, retention, and participation were major problems. Many local CBOs/FBOs who were members did not participate regularly or meaningfully. Also, the respondent provided insight regarding strained relationships with local government partners, including the LHD. The respondent described one particular disaster response in the area that was less effective than desired due to these poor relationships. 

In contrast, another VOAD representative from a different city reported an entirely different experience. First, this VOAD’s membership was highly inclusive and broad-based, but its members did not meet regularly. The general membership cannot be described as highly cohesive. However, interaction among member organizations was reported as frequent and direct (member to member) rather than using the VOAD itself as an intermediary body. Second, the VOAD representative reported a better relationship with its local government partners. The representative reported three local (small scale) emergencies where the VOAD played a key role coordinating the activities of its members in liaison with local government agencies. 

For a third example, one respondent reported her failed effort to create a VOAD in her area. In this instance, the local CBOs, FBOs, and local government agencies expressed little interest in supporting or maintaining a VOAD. However, the respondent also shared that the relationships among all local partners were sufficiently developed such that in the event of a disaster, a formal VOAD might be irrelevant to them. However, this premise has not been tested in earnest by a sufficiently large disaster. 

Finally, another area had a substitute committee that served the same functions of a local VOAD. The idea of incorporating their interaction in a different manner never occurred to the respondent because it was apparently not needed. The respondent described multiple disasters and public health emergencies within the jurisdiction in which coordination and interaction seemed to be very effective as a result of their local committee. 

### 3.5. The Not So Usual Suspects

Many LHDs seem to gravitate towards maintaining or nurturing relationships with the more visible, recognized, and/or active CBOs/FBOs in their area. With respect to disasters and public health emergencies, there are certain flagship organizations that are seen as leaders or reliable partners [6]. In some cases, this is an extension of brand recognition, prominence in the local community, or the resources they can bring to bear. We discovered several notable exceptions to these usual suspects—virtually ignored or unknown agencies that could provide substantial benefit if properly engaged by their LHDs. 

First, we discovered examples of local organizations which did not identify themselves as part of the disaster CBO/FBO community, yet they still had a great deal to offer LHDs and other partners if a relationship had existed. One such organization served a linguistically and culturally isolated minority group within its jurisdiction. The LHD primarily engaged them simply because this CBO provided them access to a hard-to-reach population. Essentially, the CBO was relegated to the role of “megaphone” for the LHD to disseminate public health preparedness and response messages. However, this particular CBO was willing and capable of providing much more had they been properly engaged and enabled, and they felt that they were not full partners with the LHD when it came to public health and emergency preparedness. For example, rather than simply disseminating messages, they believed that they could contribute by providing volunteer support (aside from translators) and making culturally relevant, tailored messages rather than simply disseminating directly translated messages on behalf of the health department. 

We found a similar organization that served the physically and developmentally disabled. This organization reported that it was completely unfamiliar with the LHD and its mission. However, the CBO had developed its own disaster preparedness activities and plans through its own initiative. Indeed, its plans were robust and well-developed in spite of its lack of contact with the LHD or other local partners normally viewed as experts in this area. They viewed themselves as more or less on their own if a disaster were to affect them or their client population, and the lack of a relationship with the LHD seemed a disservice to the larger community. 

A third example pertains to an extremely remote and isolated community. The nearest hospital was two hours away. There were no local emergency services of any kind. A local community group formed to provide a volunteer emergency response capability. They reported very different experiences working with different formal response organizations. The region’s health department had relied heavily on this group when it responded to a weather-related disaster in the area. The health department realized that since it had no local information or connections, it needed to rely on this local group for access, directions, and local knowledge. The CBO perceived value and affirmation from this interaction. However, the respondent expressed that responders representing fire, law enforcement, and emergency medical services viewed the local volunteer group as interlopers on their jurisdiction and authority. The respondent reported the response agencies as saying they did not want “amateurs” interfering in their sphere. These perceptions and interactions demeaned the CBO’s members and hurt the quality of the relationship. However, the law enforcement officials still requested support from this CBO for two separate missing persons incidents. 

### 3.6. Continuity and Consistency Matter

Inter-organizational relationships often boil down to the quality of their interpersonal relationships. Examples from our interviews provide different insights regarding the perceived importance of maintaining continuity and consistency in relationships. Without consistent interpersonal relationships, the inter-organizational relationships can suffer. 

One respondent reported that he rarely had contact with the same LHD representative for more than two consecutive meetings. It seemed to him as if the LHD rotated the responsibility of attending this particular meeting among its staff. The result was that the respondent felt as if he was starting the relationship with the LHD anew each time he met a new representative, and little got accomplished. One significant point was that the subsequent LHD representatives seemed largely unaware of prior conversations. In this instance, the respondent was referring to a LHD for a large, urbanized area. 

Another respondent reported that her LHD contact simply seemed too busy to return phone calls or emails. She reported that the contact “seemed nice”, but the contact was busy with other duties and thus nonresponsive. As a result, the FBO respondent concluded that the LHD did not seem to place importance on maintaining or improving relationships; she was very frustrated by this situation and just gave up. In this instance, the respondent was referring to an LHD for a mid-sized jurisdiction. 

Another respondent described a situation where a single employee from the LHD was its sole community outreach person. In this situation, the LHD representative was perceived as being unable to answer specific questions because she would consistently have to consult with other LHD employees for subject matter expertise. This employee was sent to various meetings for a wide range of topics, but she seemed unable to speak for the health department with any knowledge or authority for specific questions or issues. 

One respondent who represented a FBO for a rural area presented an interesting issue. Her FBO provided services across a very large geographic area that included 19 counties. As a result, she had to deal with several LHDs (not 19 LHDs, as most of the health departments covered more than one county). Still, this circumstance resulted in what she reported as strong relationships with only a handful of these LHDs with whom she had more frequent interaction (she reported that these were in areas with higher population densities). While this respondent’s situation might be less common than others, this draws attention to the fact that throughout many parts of our country, organizational jurisdictions among all parties (local government agencies, CBOs, and FBOs) hardly ever line up. Multiple, overlapping, and noncontiguous jurisdictional boundaries seem to be the norm. Indeed, in some jurisdictions, not even local fire and police district boundaries match. This issue might be worthy of separate consideration if it is deemed to present an operational problem. 

A final example might be specific to larger health departments. In this case, the respondent’s complaint was that she could never seem to get in contact with the “right” person. She reported that she was often referred to different people within the same public health emergency planning office for what she perceived to be a straightforward topic. She had to have multiple conversations to have her issue addressed, yet she had no stable, consistent relationship with any of the people who helped her. 

## 4. Discussion

The six themes identified from our analysis integrate and build to an obvious conclusion. Good inter-organizational relationships depend on the underlying interpersonal relationships of their organizational representatives. Across different organizational and jurisdictional characteristics, our respondents describe the importance of and challenges presented by relationships from their perspective. On one hand, it is apparent that the relationship challenges as they relate to disasters among LHDs-CBOs/FBOs are fundamentally no different than the inter-relationship challenges in other organizational contexts. These are very common group dynamics and conflict issues. However, we also indentified that the interviewed CBO/FBO representatives seemed largely unaware of commonly used relationship building and conflict resolution strategies. Furthermore, there was no evidence from our interviews that LHDs were actively employing such strategies. In most cases, the CBO/FBO representatives intimated that their intergroup relationship problems were unique and exceptional to their situation rather than normal and even common to other groups.

Notably, the articles cited for background draw very little if anything from the extensive bodies of organizational and social psychology literature that speak to intergroup dynamics. Indeed, there are exceedingly few examples of research that applies an intergroup dynamics approach to the collaboration of organizations active in disaster (quite stunning, given the intergroup nature of these collaborations). This might be due to silos that separate academic disciplines, but at face value, it is clear that not all available theoretical and analytical tools are being applied to intergroup relations as they pertain to disasters. 

Seminal examples from group and intergroup studies include Tuckman’s stages of group development and Alderfer’s exploration of intergroup theory [32,33,34]. From the perspective of Tuckman’s stages of group development, the VOAD member organizations could avoid the preliminary stages of group “forming, storming, and norming” [32,33]. They would have already passed through those stages as a result of their regular contact through the VOAD, and they could thus move straight to “performing” (effective group function). The group would already be established and have passed through the earlier formative stages. The same is true for contributions to our understanding of intergroup conflict [35,36]. To presume that group and intergroup dynamics around the topic of disasters are immune to the same dynamics that affect all other groups (especially those under stress) suggests an enormous theoretical blind spot. Indeed, numerous scholarly articles have sprung from the basic theories presented by the founders of these disciplines. 

In spite of this, there are examples where our findings do fit with the literature. For example, Kapucu found that CBOs were necessary to the 9/11 response in New York, and better pre-disaster relationships among local government agencies and CBOs/FBOs could have streamlined their role in the response [28]. Forming relationships after the disaster is less preferable than exercising relationships that have been established beforehand. Kapucu also found that the repeated threat of imminent hurricanes in Florida led to closer relationships and more effective collaboration among local government agencies and CBOs/FBOs [27]. This corresponds to the conclusion drawn by Paige *et al*. regarding the benefits to relationships through joint exercises [26]. 

Nothing presented in this article is meant to be an indictment of LHD public health emergency planners. We acknowledge several possible reasons for perceptions of unsatisfactory LHD-CBO/FBO relationships with regard to disasters and public health emergencies. First, the modern explosion of public health emergency funding, planning, and programs is barely a decade old (post-Anthrax attacks and SARS). Public health professionals are relatively new to formal participation in emergency management when compared to law enforcement and fire service professionals. As such, it also stands to reason that public health emergency planners may have not yet fully asserted themselves as equal players in this sphere who can operate in a way that suits their strengths. 

While the emergency management environment requires multi-agency collaboration, the organizations which have traditionally dominated this environment (e.g., law enforcement and fire service) are characterized by their hierarchical, “command and control”, top-down management paradigm [37]. It is thus quite arguable that the management culture of the organizations that have traditionally taken leadership roles in emergency management has permeated the nature of emergency management collaborations. Thus, public health emergency planners seem to be operating in an environment heavily influenced by a management culture that is quite different from the public health management culture and structure, which is generally thought of as more collaborative and horizontal/lateral than how the uniform-wearing “first responder” community seems to function [38]. 

We believe that this apparent clash of cultures might be influencing the relationships that public health emergency planners have with CBOs/FBOs, especially when some of those same CBOs/FBOs have prior experience working with LHDs in a different context and manner. In fact, we are not alone in that belief. Although the collaborators in question were different actors, the essential nature of this observation was central to a 2008 framework developed by the Public Health and Law Enforcement Emergency Preparedness Workgroup, a collaboration between the CDC and the U.S. Department of Justice. Organizations that collaborate across sectors for disasters have different organizational cultures, philosophies, and operating methods. This document promotes the idea that cross-sector collaborations are best improved through proactive steps to engage partners, and the gap represented by their cultural differences can be reduced by joint activities that require/facilitate cooperative action [39]. This suggests that actively working together is the best prescription for improving inter-organizational relationships and collaboration even when the partners have meaningful differences in culture or philosophy. 

Our study follows closely behind a 2011 CDC publication, *Principles of Community Engagement* (Second Edition) [40]. It provides guidance for public health professionals regarding community partnerships, and it is clear that some of the findings from our study suggest that public health emergency planners might benefit by familiarizing or reacquainting themselves with this document. Our study confirms the need for this document, which provides theoretically-based and tested methods for effectively partnering with community organizations. While it is clear that there will always be an inevitable gap between any theoretical ideal and the resource-constrained pragmatism in the field, the principles in this document are worth aspiring towards. If nothing else, this study reaffirms many of the principles presented in this CDC document. For example, the text advises LHDs to “make no assumptions about the capabilities of the [CBO] or how it functions” [40]. This relates to the issue of expectation management introduced earlier. 

This also relates to MOUs. While MOUs in different contexts can express a wide range of specificity and commitment, in many cases they are essentially written agreements to respect the independence and autonomy of the signatories. Most stipulations refer to issues culturally common (or mandated) among many organizations, such as non-discrimination clauses. LHDs can mitigate any reluctance that CBOs or FBOs might have regarding MOUs if they first invest the time in establishing interpersonal relationships and introduce the subject of MOUs in a different manner. 

When it comes to the availability of and access to resources, it seems that there will generally be fewer resources available than desired under ideal circumstances. We were presented with the notion that relationships are sought or developed because of resources that can be gained through them (6). Our evidence does not support this argument (from the CBO/FBO perspective, at least). In fact, most of our CBO/FBO respondents asked for or needed little in the way of discrete resources from their LHD counterparts. Some of them could not even identify what they would ask for if the opportunity for resources was presented to them. The issues were more focused on staff time, communication, respect, trust, and governance. 

The CBOs/FBOs seemed more interested in honest and meaningful relationships than in funding or supplies. Our findings suggest that LHDs can build relationships and facilitate CBO/FBO capacity building through “soft” resources (e.g., staff contact time or access to training or meetings that are already being conducted). Any reported dissatisfaction or conflicts reported by the CBOs/FBOs had less to do with “what” (*i.e.*, the allocation or sharing or discrete resources) and more to do with “how”. Relationships matter more than materiel, and CBOs/FBOs can be greatly empowered with the benefit of a few simple resources shared through meaningful relationships. 

Of course, staff time is perhaps an LHD’s most critical resource. To be fair, LHDs cannot be expected to devote a significant amount of additional staff time to developing and maintaining relationships unless it is funded to do so or unless it takes staff time from other tasks. The same is true for the CBOs and FBOs. In that light, the issue turns to how to use the available staff time more effectively. Expressing the opposing viewpoint presented by this study’s respondents, Varda *et al.* qualitatively examined the impact of CBO/FBO collaboration on LHD staff time from the perspective of LHD respondents [6]. They recommended that LHDs minimize the staff-time impact of collaborations by forming fewer but more strategic partnerships, and they further recommended that collaboration be seen as a means to an end rather than a worthwhile end in itself. Their findings challenge the underlying presumption of the CDC and FEMA documents cited earlier, which link effective community collaborations to better outcomes from a “best practices” perspective (not backed up by quantitative research) [18,39,40]. This leads to a discussion of how to form strategic relationships to maximize outcomes while minimizing the cost in terms of staff time. Even the presumed panacea of joining or creating a VOAD is not what it initially seemed to be. 

The situation is more complicated than whether or not a VOAD (or equivalent organization) is present or active. A separate inquiry needs to be made regarding the variation among VOADs in terms of their organizational structure, membership, management techniques, and the local social landscape. It is a matter of how well the local VOADs function rather than whether or not they exist. There is meaningful and under-studied variation regarding how VOADs develop and operate wherever they exist (in spite of their shared principles and guidelines). They are all very different, primarily as a function of the local leadership, their business model, and community characteristics. Some are well-managed; some are not. Some have good relationships with their local government agencies; some do not. Some have broad membership or association representing a wide range of CBOs/FBOs; some have narrow and/or exclusive membership (thus making them more insular). There appear to be other meaningful dimensions of variation among VOADs. 

Our initial premise was that if a VOAD was present in the local jurisdiction, then a LHD’s regular interaction with CBOs/FBOs through the VOAD would universally improve the strength and quality of their partnerships with the VOAD’s member organizations (while reducing the impact on staff time). As a corollary, we could have argued that if a local VOAD (or equivalent organization) did not exist, then the LHD would be well advised to create and support a VOAD. Two words seem to have derailed these hopeful premises: “local politics”. VOADs and their characteristics are a direct outgrowth of the local social fabric. The manner in which a VOAD organizes itself and functions might really be about finding a good “fit” for the local characteristics; what works for one VOAD might likely not work for another. From a researcher’s perspective, the presence of a VOAD creates more questions than it answers with regard to its impact on intergroup relations, especially with the LHD. 

In general, our interviews suggest three steps that can help LHDs move forward. First, they can invest time in honestly and accurately assessing the quality of relationships with their CBOs/FBOs. Second, they can actively reach out to CBOs, FBOs, and VOADs to create new partnerships (or to renew them). Third, they can train their staff to employ commonly-used intergroup dynamics and conflict resolution skills. These steps can provide LHDs with a better foundation from which they can improve the breadth and depth of their community relationships (and the subsequent benefit of these relationships). The keys are meaningful, consistent, and ongoing interaction and participation. If LHDs agree with the notion that they can better perform their own missions through effective CBO/FBO partnerships (as multiple government documents urge them to realize), then it would also be in their interest to elevate the importance of relationship building and maintenance as a priority for their public health emergency planners. 

Local health departments clearly face myriad challenges, but it is in their interest to know what it is like for a community organization representative to work with them. It is up to each LHD to organize itself and allocate its constrained resources as best as it can, but they need to understand how this affects their relationships with CBOs/FBOs trying to work with them. It seems that maintaining stable and consistent relationships deserves higher priority. In the end, the trade-offs presented by constrained resources will overcome the best of intentions, but perhaps health departments can better understand and address or mitigate these trade-offs with consideration to how it affects their relationships with their CBOs/FBOs. 

## 5. Conclusions

As reported by our key informants, the breadth and depth of their relationships with local LHDs was more often than not relegated to the role of information dissemination. Many LHDs were described as using a one-way “push” model with many of our CBOs/FBOs rather than engaging them in other ways as partners. The relationships, such as they were, were self-limiting. While we are definitely sympathetic to understaffed and overworked local health departments, our findings suggest that the LHDs were actually hurting themselves in those cases where relationships were less meaningful or less appreciated from the perspective of the CBOs/FBOs. 

If LHDs invest the time to create partnerships with CBOs/FBOs on public health emergency preparedness, the return on investment is that the LHDs will actually be able to do their own jobs better and serve their communities more effectively as a result of these relationships. The irony, of course, is that for many other public health functions, LHDs have demonstrated exemplary partnerships. For some reason, the CBOs/FBOs in our sample indicate that LHDs have not been able to extend their otherwise excellent partnership skills to the specific function of public health emergency preparedness. This irony is not fully understood, but it could be an extension of the idea that the PHEP field operates less like other public health programs and more like leading organizations within the emergency management community (*i.e.*, formal, hierarchical, and uniformed services). This issue is worthy of further exploration. 

In crisis situations, we know that various actors are more likely to exercise relationships that already exist rather than create new relationships under emergent circumstances. In those cases where relationships are formed during a crisis, group processes and outcomes are often plagued by problems. Thus, it follows that PHEPs need to be embedded and well-positioned in their community’s CBO/FBO networks prior to any emergency or disaster. A network position/structure that has an effective balance of strong and weak organizational ties will allow the LHD PHEPs the optimum level of capacity, reach, and flexibility to react to emergent circumstances. Whether or not a formal VOAD exists in the jurisdiction is less important than whether or not the VOAD is effective and healthy. More exploration into the variation among VOADs and their effectiveness would be helpful for understanding this better. 

Regarding many of these issues, the answer boils down to leadership. LHDs are certainly not guilty of not being busy enough. We understand that they are often overworked and trying to manage multiple, competing priorities given their own constrained resources. The type of leadership we are calling for is based in the belief that better attention to CBO/FBO relationships on the subject of public health emergency preparedness will actually help LHDs do their own job better. Recommendations to LHD PHEPs, as derived from our key informants, include several things that may seem difficult, but the return on investment could be substantial when most-needed. 

Our findings suggest that the creation and maintenance of relationships should receive higher priority in terms of staff time. Furthermore, professional development training in the form of intergroup dynamics and conflict resolution skills might prove helpful. Another suggestion is to conduct a thorough and honest assessment of the quality of the LHD’s relationships to CBOs/FBOs. While discrete resources are always desirable, it’s more important to devote staff time towards building relationships that communicate respect and value for all CBO/FBO partners. Through helping them develop capacity as a result of these relationships, the LHD will ultimately realize that their own response to disasters or public health emergencies will be easier. 

We have also raised several other points throughout this study that are worthy of further research. Most significantly, we were unable to find any academic research which examined the organizational culture of public health organizations in comparison to their emergency management, law enforcement, and fire service counterparts. The differences seem to have face validity, but this should be studied. We were intrigued by the implication that public health emergency planners might largely be thrust into an environment heavily influenced by a different organizational culture and structure. Further academic inquiry might better explain this apparent dissonance, as well as how it might account for why public health professionals seem to work better with CBOs/FBOs in more traditional public health programs (e.g., nutrition or vaccinations) than they do with public health emergency planning. We urge a thoughtful and explicit adaptation of basic organizational and social psychology theories to the subject of CBOs and FBOs active in disasters. 

Our research suggests the need for a more meaningful understanding of how various organizational and geographic variations might influence inter-organizational relationships and collaborations. This study’s sample purposefully selected CBO/FBO representatives from a broad range of organizational sizes, missions, capabilities, and differences in the populations and jurisdictions that they served. Further studies can quantify and elucidate the meaning of the variations we have introduced. Finally, as introduced by Varda *et al.*, we see tremendous promise in Social Network Analysis as a method for properly diagnosing and improving inter-organizational relationships [6]. 

Our study has several limitations that are important to note. First, it is a qualitative study, meaning that its results are by definition not meant to be externally valid or generalizable. The six themes and their implications are meant to guide further inquiry. In essence, we have revealed questions rather than answered them. Second, our sample was purposive and limited in size. Future studies seeking to better illuminate the themes we have identified can select samples in a way that best suits their study design. As we were trying to maximize the diversity or organizational characteristics represented in our sample, future studies might use samples and designs that allow for bivariate analysis based on clearly identified organizational characteristics (size, mission, nature of jurisdiction, *etc.*). Our study is not able to draw generalizable or externally valid results based on these variations. 
